# Giant well-differentiated liposarcomas of the gluteal region: A case report

**DOI:** 10.1097/MD.0000000000046788

**Published:** 2026-01-09

**Authors:** Futao Ji, Junpu Luo, Liuhui Wang, Pengcheng Yao, Liubin Lu, Guoqing Li, Wei Liang, Kai Zhang

**Affiliations:** a Orthopedic Center, Zhengzhou 460 Hospital, Zhengzhou, China.

**Keywords:** clinical pathology, diagnosis, gluteal region, well-differentiated liposarcoma

## Abstract

**Rationale:**

Well-differentiated liposarcoma (WDL), a rare mesenchymal tumor with adipocytic differentiation, represents a distinct subtype of liposarcoma (LPS). It demonstrates relatively high incidence and typically arises in the deep soft tissues of the extremities, retroperitoneum, or cervical regions. This case report presents a 55-year-old female patient with a 12.0 cm × 9.0 cm × 6.5 cm tumor in the right gluteal region, which was successfully managed with surgical resection in the absence of distant metastasis.

**Patient concerns:**

A 55-year-old female patient was admitted to our hospital due to right gluteal swelling accompanied by progressive ipsilateral lower limb soreness and numbness for 3 days, which worsened with activity and alleviated at rest, leading to difficulty in walking. Based on clinical manifestations, physical examination, and imaging findings, a preliminary diagnosis of gluteal liposarcoma was considered.

**Diagnoses:**

Histopathological analysis definitively diagnosed a giant WDL located in the right gluteal region.

**Interventions:**

The lesion was completely removed with marginal resection, achieving en bloc excision along the plane outside the tumor capsule.

**Outcomes:**

The patient was discharged on postoperative day 14 without complications. Telephone follow-up at 1, 3, 6 months, and 1 year postoperatively, along with ultrasound and magnetic resonance imaging examinations, revealed no evidence of recurrence.

**Lessons:**

WDL represents a rare neoplastic entity. Due to its nonspecific clinical presentation and diagnostic inexperience, definitive preoperative diagnosis remains challenging. Accurate diagnosis of WDL relies on comprehensive histopathological examination, immunohistochemical profiling, and molecular analysis. Our findings underscore the critical importance of surgical intervention to prevent neurovascular complications and optimize clinical outcomes.

## 1. Introduction

Lipomas represent the most prevalent soft tissue neoplasms, affecting approximately 1% of the general population.^[1,2]^ According to the World Health Organization (WHO) Classification of Soft Tissue Tumors (5th edition), liposarcomas are histologically categorized into 5 principal subtypes: well-differentiated, dedifferentiated, myxoid, pleomorphic, and myxoid pleomorphic types. Among these, well-differentiated liposarcoma (WDL)/atypical lipomatous tumor (ALT) represents the most prevalent subtype. However, due to diagnostic challenges and limited clinical experience, establishing a definitive preoperative diagnosis remains difficult, often delaying accurate and timely therapeutic interventions.Although imaging modalities provide valuable diagnostic assistance, they exhibit inherent limitations in precisely characterizing the disease nature. In the gluteal region, WDL and ALT demonstrate overlapping clinical presentations with remarkably similar symptomatology and physical findings, rendering clinical differentiation particularly challenging. Consequently, complete surgical excision is mandatory, with definitive diagnosis requiring comprehensive postoperative histopathological evaluation supplemented by immunohistochemical studies and molecular analysis.^[3]^

## 2. Case description

A 55-year-old Chinese female presented to the orthopedic department with a 3-day history of right gluteal swelling accompanied by progressive ipsilateral lower extremity soreness and numbness. The predominant symptoms included right gluteal distension, tenderness, and numbness, exacerbated by physical activity and alleviated in the supine position, resulting in ambulatory difficulty. No prior therapeutic interventions or surgical history were documented. The patient denied relevant familial or social medical history.

**Figure 1.**
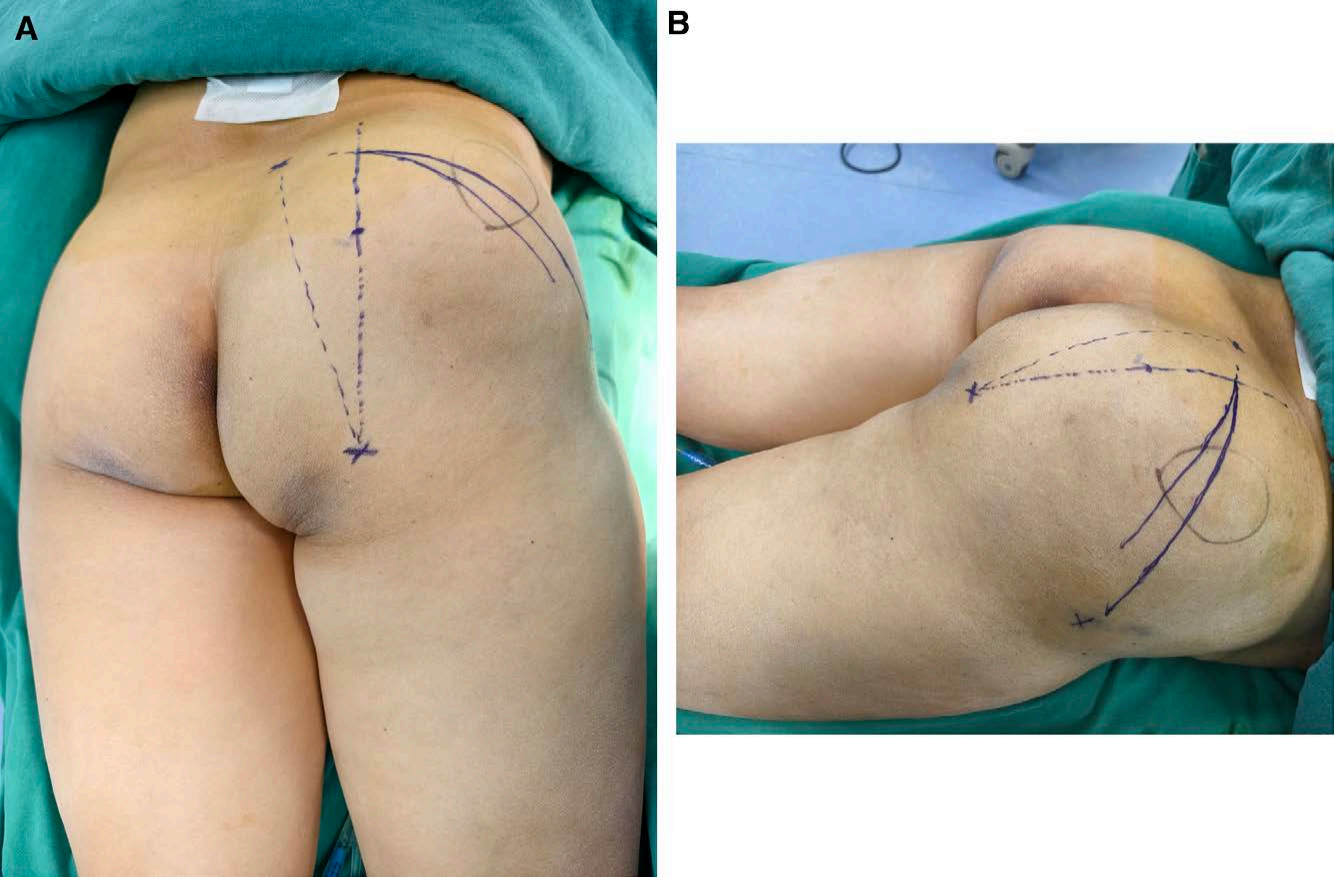
(A and B) Preoperative buttocks presentation.

Physical examination revealed significant diffuse swelling over the right buttock with a palpable well-circumscribed mass demonstrating tenderness on compression. Positive femoral nerve traction test and Faber test were noted (Fig. 1A and B).

**Figure 2.**
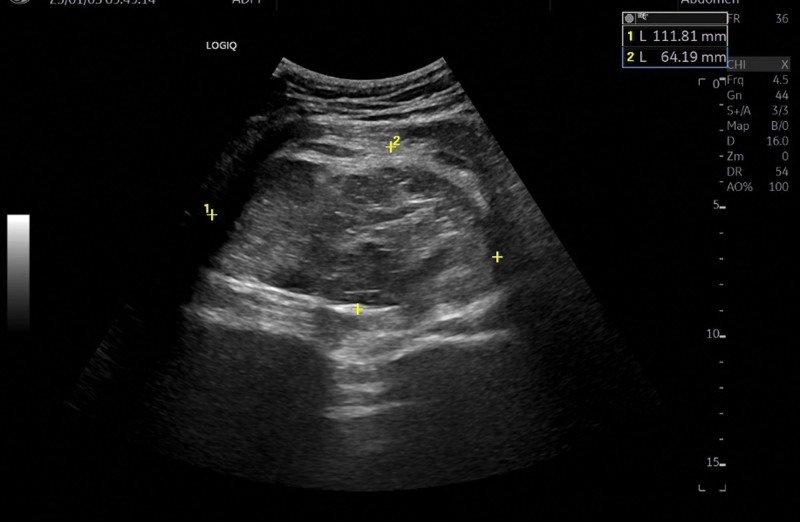
MSK-US. The MSK-US identified a 12.8 cm × 8.1 cm × 11.7 cm lobulated mass with regular margins and absence of vascular flow signals, suggestive of a solid lesion anterior to the piriformis muscle (recommended further investigation). MSK-US = musculoskeletal ultrasound.

**Figure 3.**
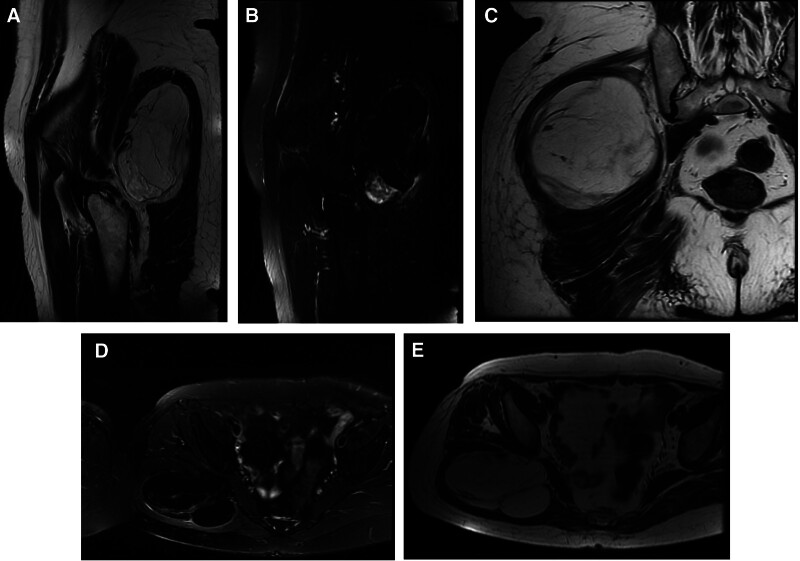
(A–E) MRI. On MRI (T1/T2-weighted sequences in coronal, sagittal, and axial planes), a well-defined gluteal mass measuring 10.37 × 10.55 × 6.2 cm (LR × SI × AP) was identified, demonstrating compression of neighboring muscular soft tissue structures. AP = anterior–posterior, LR = left–right, MRI = magnetic resonance imaging, SI = superior–inferior.

Initial lumbar spine radiography showed no osseous abnormalities, prompting further evaluation with musculoskeletal ultrasound of the right hip. The musculoskeletal ultrasound identified a 12.8 cm × 8.1 cm × 11.7 cm lobulated mass with regular margins and absence of vascular flow signals, suggestive of a solid lesion anterior to the piriformis muscle (recommended further investigation) (Fig. 2). Subsequent magnetic resonance imaging demonstrated a fat-containing soft tissue mass within the right gluteus maximus, raising suspicion for liposarcoma (Fig. 3A–E). Due to the indeterminate radiological features, definitive diagnosis requires histopathological examination of tissue specimens to achieve reliable diagnostic confirmation.

**Figure 4.**
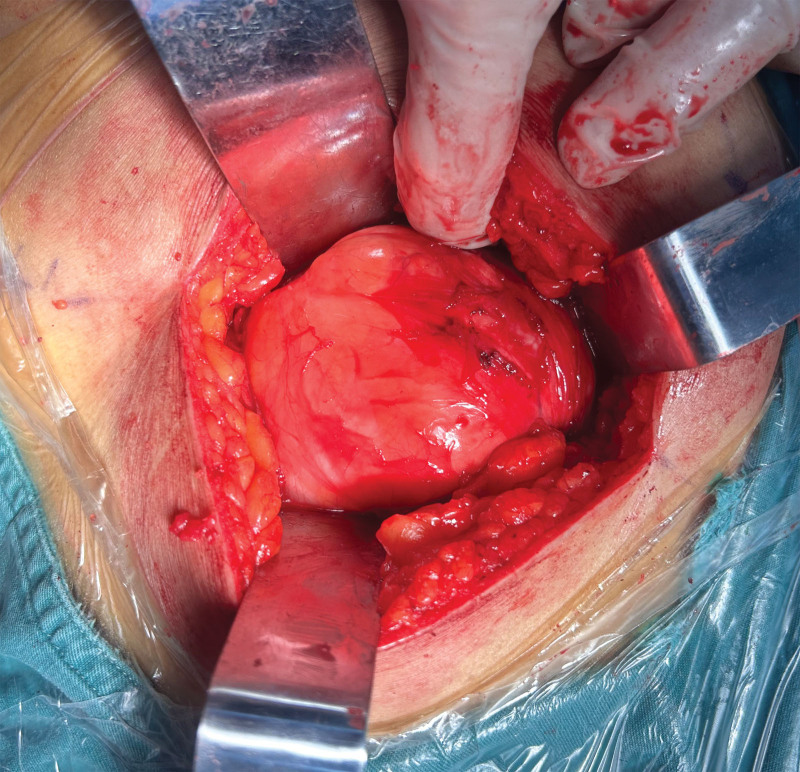
Intraoperative exploration revealed a giant encapsulated intramuscular mass occupying the majority of the gluteal compartment.

**Figure 5.**
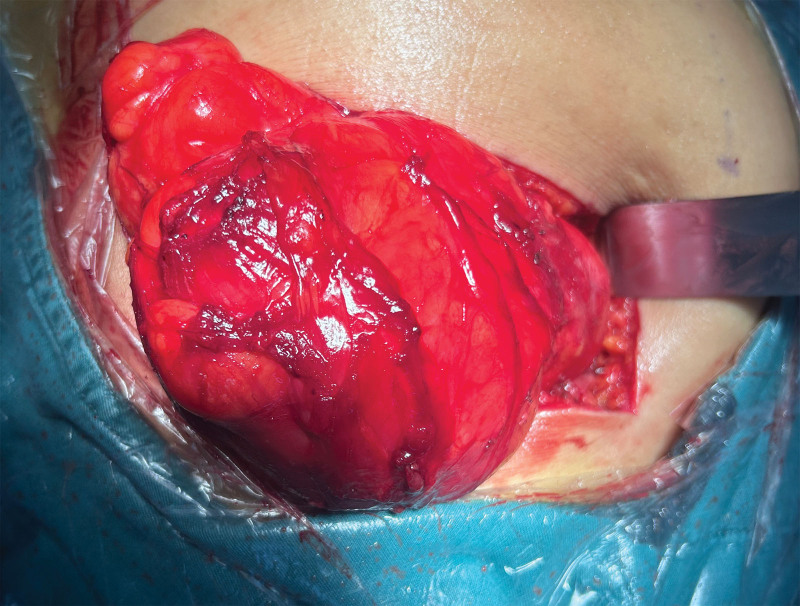
Complete exposure of the tumor during surgery.

**Figure 6.**
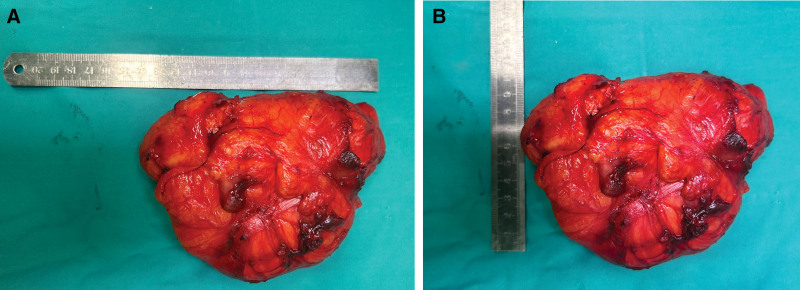
(A and B) Gross photo of postoperative specimen.

Following informed consent, the patient underwent marginal resection with complete excision along the pericapsular plane. Intraoperative exploration revealed a large, well-encapsulated mass occupying the majority of the right gluteal region (Fig. 4). Blunt dissection through the gluteus maximus muscle exposed a smooth tumor capsule covered by normal overlying tissue (Fig. 5). Retraction of the gluteus medius muscle demonstrated significant compression of the underlying piriformis muscle. Meticulous dissection maintained complete capsular integrity, achieving en bloc resection with tumor-free margins. Intraoperative neurological assessment confirmed preserved sciatic nerve integrity with normal tension (Fig. 6A and B).

**Figure 7.**
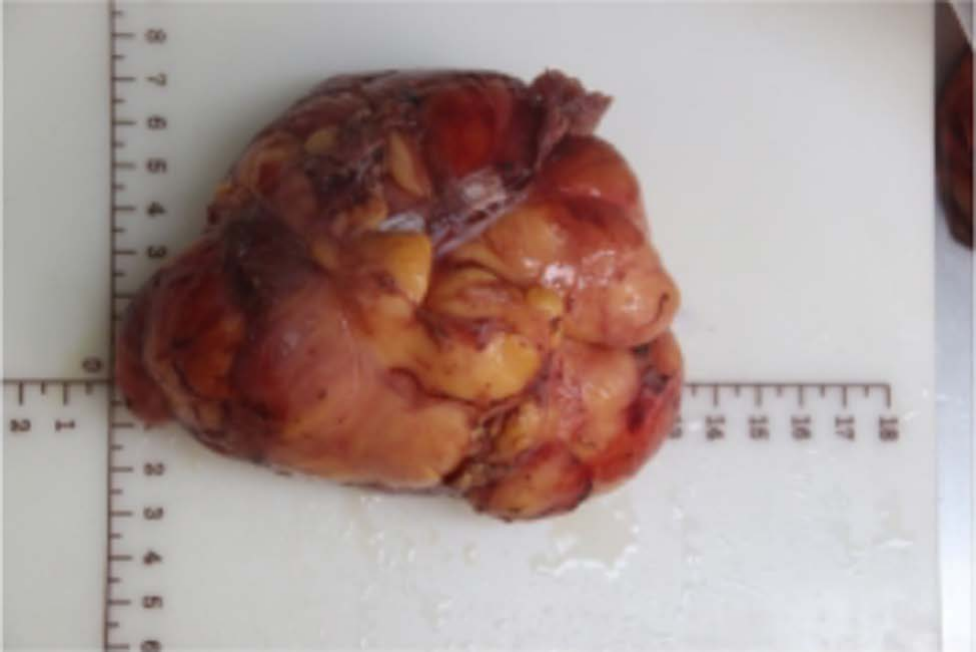
Postoperative examination photos.

**Figure 8.**
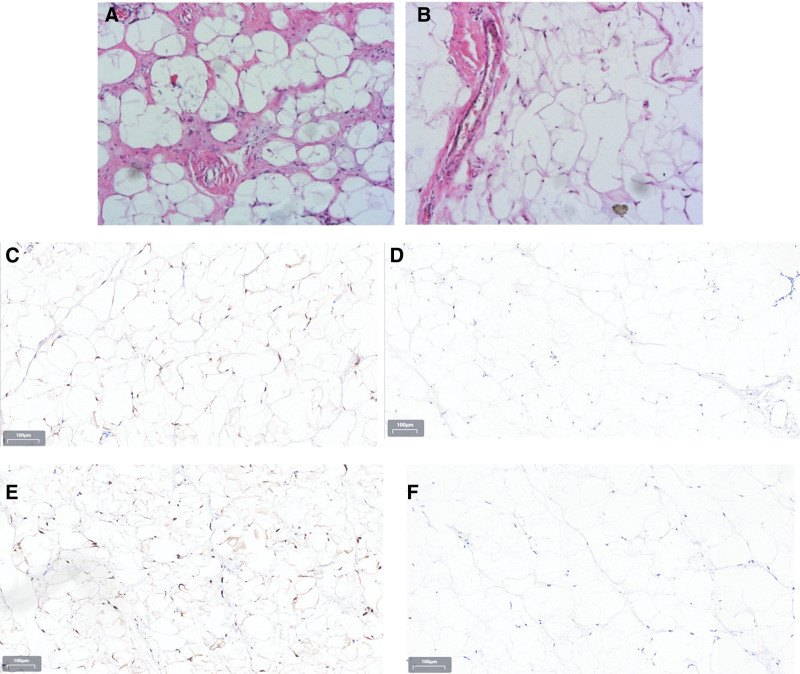
Histopathological, immunohistochemistry, and molecular analysis of tumors. Histopathological analysis revealed that the tumor was centered on the superior subcutaneous stroma and lined with intact squamous mucosa (HE × 100). (A and B) Tumors are composed of mature adipocytes and some atypical, enlarged spindle cells with hyperstained nuclei (A: HE × 100 and B: HE × 100). (D and F) immunohistochemical staining showed that tumor cells were negative for Ki-67 and MDM2 (D: HE × 100 and F: HE × 100), and (C and E) immunohistochemical staining showed that tumor cells were positive for P16 and CDK4 (B: HE × 100 and D: HE × 100).

**Figure 9.**
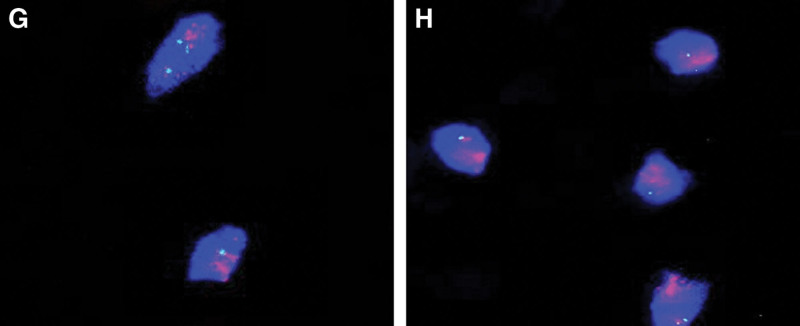
MDM2 and CDK4 gene amplification (red signal) was confirmed by fluorescence in situ hybridization (FISH) analysis. (G and H) FISH(MDM2): the MDM2/CEP12 probe set comprises 2 DNA probes that generate bright, microscopically visible signals upon hybridization to both metaphase chromosomes and interphase nuclei. The MDM2 DNA probe hybridizes to the long arm of human chromosome 12 (12q15) and produces a red fluorescent signal. The control probe, CEP12, targets the pericentromeric region of chromosome 12 (12p11.1-q11.1), covering the entire centromeric area, and emits a green fluorescent signal. FISH(CDK4): the CDK4/CEP12 probe set consists of 2 DNA probes that generate bright, microscopically detectable signals upon hybridization to both metaphase chromosomes and interphase nuclei. The CDK4 DNA probe hybridizes to the long arm of human chromosome 12 (12q14) and produces a red fluorescent signal. The control probe, CEP12, targets the pericentromeric region of chromosome 12 (12p11.1-q11.1), encompassing the entire centromeric area, and emits a green fluorescent signal.

*Pathological findings*: The resected specimen measured 12.0 cm × 9.0 cm × 6.5 cm and weighed 2.5 kg (Fig. 7). Light microscopic examination revealed fibroadipose tissue consistent with lipoma (Fig. 8A and B). Immunohistochemical staining demonstrated tumor cell positivity for P16 (Fig. 8C) and CDK4 (Fig. 8E), while being negative for Ki-67 (Fig. 8D) and MDM2 (Fig. 8F). Furthermore, fluorescence in situ hybridization analysis confirmed gene amplification involving both MDM2 (Fig. 9G) and CDK4 (Fig. 9H).

Based on these findings, the diagnosis was well-differentiated liposarcoma of the buttock. The patient received anti-infection, hemostatic, and fluid replacement therapy. Follow-up evaluations via telephone and imaging (ultrasound and magnetic resonance imaging) at 1, 3, 6 months, and 1 year postoperatively showed no signs of complications or local recurrence.

## 3. Discussion

WDL is a low-grade soft tissue sarcoma with a propensity for local recurrence. Liposarcomas typically arise in adipose-rich regions such as the extremities, abdominal cavity, and retroperitoneal space. Although they often exhibit expansive growth, their progression is relatively slow compared to other malignancies. However, studies indicate that tumor growth rates may vary at different stages.^[4,5]^ Among the subtypes of liposarcoma, ALT/WDL is the most common, accounting for approximately 40% of all liposarcomas.^[6]^ WDL demonstrates locally aggressive behavior and may undergo dedifferentiation into a higher-grade sarcoma (i.e., dedifferentiated liposarcoma), which carries a significant metastatic potential. Given the distinct biological characteristics of these lipomatous tumors, accurate diagnosis is essential for optimal management.^[7]^

Based on clinical experience, WDL rarely occurs in superficial soft tissues. It typically presents as a painless, slow-growing mass that can reach considerable dimensions. In buttock locations, WDL may be incidentally detected during radiographic examinations, with reported diameters ranging from 2.0 to 35 cm (median: 18 cm).The most significant prognostic factor is its anatomical location. Local recurrence rates for extremity liposarcomas show considerable variation in the literature (8–52%). The median time to local recurrence is 48 months from the date of surgery.^[8–10]^

The nonspecific clinical manifestations of WDL pose significant challenges for timely and accurate diagnosis. While imaging studies provide valuable diagnostic assistance, they exhibit inherent limitations in definitively characterizing the lesion preoperatively. In the present case, radiological examinations revealed a fatty soft tissue mass within the right gluteus maximus muscle, initially suggestive of liposarcoma. However, the imaging features proved insufficient for reliable differentiation from other adipocytic tumors, particularly lipoma, potentially leading to diagnostic misinterpretation. Consequently, definitive diagnosis remained dependent on postoperative histopathological evaluation. The combined immunohistochemical staining panel of p16, MDM2, and CDK4 serves as a crucial tool for distinguishing WDL from ALT and other adipocytic neoplasms, including conventional lipoma.^[11]^ Current evidence demonstrates that detection of MDM2 and/or CDK4 gene amplification represents the most reliable method for differentiating WDL from benign lipomas, offering both high sensitivity and specificity.^[12]^ Notably, in our case, fluorescence in situ hybridization analysis confirmed concurrent CDK4 and MDM2 gene amplifications.The final diagnosis of gluteal well-differentiated liposarcoma was conclusively established through comprehensive postoperative assessment incorporating histopathological examination, immunohistochemical profiling, and molecular genetic analysis.

Complete surgical resection serves as the cornerstone of radical treatment, effectively removing the tumor to alleviate symptoms while achieving curative intent. The surgical approaches vary, primarily encompassing wide excision and marginal excision. Chang et al demonstrated that the wide excision group had a higher incidence of postoperative complications compared to the marginal excision group, while both groups exhibited similar local recurrence rates. When tumors are adjacent to functionally critical structures such as blood vessels or nerves, wide excision may lead to severe surgical complications.^[13]^ Furthermore, Olson et al found that microscopically positive margins were not associated with an increased risk of local recurrence.^[14]^ However, a recent systematic review revealed that the marginal excision group did not show a significantly elevated local recurrence rate.^[15]^ Given the tumor’s well-defined capsule and clear boundaries, planned marginal excision is a feasible approach.

In this case, the patient presented with a well-demarcated WDL. As the mass progressively enlarged, she developed ipsilateral gluteal swelling and localized pain, accompanied by sensory numbness and lower extremity symptoms, which prompted her to seek medical intervention. Postoperatively, her symptoms resolved completely following en bloc resection of the lesion.

This study is limited by short follow-up period, which may not fully capture long-term recurrence patterns.

## 4. Conclusion

This case report describes a rare giant WDL arising in the gluteal region. Due to its nonspecific clinical presentation and the limited diagnostic experience associated with such cases, establishing a definitive preoperative diagnosis remains challenging despite suggestive clinical and imaging findings. The gold standard for definitive diagnosis relies on histopathological examination supplemented by immunohistochemical staining (e.g., MDM2 and CDK4) and molecular analysis. Currently, surgical resection with marginal margins represents the mainstay of treatment, and early detection combined with complete tumor excision is critical for minimizing the risk of local recurrence.

## Acknowledgments

We are grateful to the medical personnel who were caring for the patient.

## Author contributions

**Conceptualization:** Futao Ji, Junpu Luo, Liuhui Wang, Pengcheng Yao, Liubin Lu, Guoqing Li, Wei Liang.

**Funding acquisition:** Junpu Luo, Wei Liang.

**Investigation:** Futao Ji, Junpu Luo, Liuhui Wang, Pengcheng Yao, Liubin Lu, Guoqing Li, Wei Liang.

**Methodology:** Futao Ji, Junpu Luo, Liuhui Wang, Pengcheng Yao, Liubin Lu, Guoqing Li, Wei Liang.

**Project administration:** Pengcheng Yao, Liubin Lu, Guoqing Li.

**Writing – original draft:** Futao Ji.

**Writing – review & editing:** Kai Zhang.
